# Glycine-Conjugated Bile Acids Protect RPE Tight Junctions against Oxidative Stress and Inhibit Choroidal Endothelial Cell Angiogenesis In Vitro

**DOI:** 10.3390/biom11050626

**Published:** 2021-04-23

**Authors:** Cassandra Warden, Milam A. Brantley

**Affiliations:** Vanderbilt Eye Institute, Vanderbilt University Medical Center, Nashville, TN 37232, USA; cassandra.warden@vumc.org

**Keywords:** age-related macular degeneration, bile acids, angiogenesis, RPE, choroidal endothelial cell, GCA, GDCA, GUDCA

## Abstract

We previously demonstrated that the bile acid taurocholic acid (TCA) inhibits features of age-related macular degeneration (AMD) in vitro. The purpose of this study was to determine if the glycine-conjugated bile acids glycocholic acid (GCA), glycodeoxycholic acid (GDCA), and glycoursodeoxycholic acid (GUDCA) can protect retinal pigment epithelial (RPE) cells against oxidative damage and inhibit vascular endothelial growth factor (VEGF)-induced angiogenesis in choroidal endothelial cells (CECs). Paraquat was used to induce oxidative stress and disrupt tight junctions in HRPEpiC primary human RPE cells. Tight junctions were assessed via transepithelial electrical resistance and ZO-1 immunofluorescence. GCA and GUDCA protected RPE tight junctions against oxidative damage at concentrations of 100–500 µM, and GDCA protected tight junctions at 10–500 µM. Angiogenesis was induced with VEGF in RF/6A macaque CECs and evaluated with cell proliferation, cell migration, and tube formation assays. GCA inhibited VEGF-induced CEC migration at 50–500 µM and tube formation at 10–500 µM. GUDCA inhibited VEGF-induced CEC migration at 100–500 µM and tube formation at 50–500 µM. GDCA had no effect on VEGF-induced angiogenesis. None of the three bile acids significantly inhibited VEGF-induced CEC proliferation. These results suggest glycine-conjugated bile acids may be protective against both atrophic and neovascular AMD.

## 1. Introduction

Age-related macular degeneration (AMD) remains a leading cause of irreversible vision loss in older adults, despite currently available treatments. In advanced AMD, vision loss results from either geographic atrophy (GA), in which photoreceptors and underlying retinal pigment epithelium (RPE) degenerate, or from neovascular AMD (NVAMD), in which blood or serous fluid leaks from abnormal choroidal vessels. Neovascularization occurs when choroidal endothelial cells (CECs) migrate through the blood retina barrier (BRB), proliferate, and form tubes of new vessels. There is currently no effective treatment for GA, and while intravitreal injections of anti-vascular endothelial growth factor (anti-VEGF) medications are routinely used to treat NVAMD, they are expensive, and the need for ongoing treatment is a significant burden on patients. It is therefore important to investigate mechanisms of AMD susceptibility and progression to discover earlier opportunities to intervene and preserve vision.

Bile acids have been shown to exert beneficial effects in experimental models of retinal diseases. The primary bile acids cholic acid (CA) and chenodeoxycholic acid (CDCA) are synthesized from cholesterol in the liver, and gut microbiota produce secondary bile acids via hydroxylation, deconjugation, epimerization, or oxidation. Primary and secondary bile acids are both commonly conjugated to glycine or taurine in the liver. In addition to emulsifying lipids to promote their absorption during digestion, bile acids are now widely recognized as signaling molecules, modulating diverse metabolic processes such as glucose and lipid metabolism [1]. The bile acids ursodeoxycholic acid (UDCA) and tauroursodeoxycholic acid (TUDCA) have been shown to protect against photoreceptor cell loss in multiple models of inherited retinal degeneration [2,3,4,5,6]. More recently, studies have investigated potential protective roles for UDCA [7] and TUDCA [8,9] against the development and progression of diabetic retinopathy in rodent models. However, few studies have examined bile acids in RPE cells or in CECs, the two cell types most prominently involved in AMD pathophysiology. One recent study reported that TUDCA protected against oxidative damage in a transformed human RPE cell line [10]. TUDCA has also been shown to inhibit human retinal endothelial cell proliferation in vitro [11] and suppress laser-induced choroidal neovascular membrane formation in rats [12].

Our laboratory recently conducted two independent metabolomics studies demonstrating that plasma levels of glycine- and taurine-conjugated bile acids were altered in NVAMD patients compared to controls, suggesting that bile acid metabolism may be relevant to AMD pathophysiology [13,14]. We then demonstrated in vitro that the conjugated primary bile acid taurocholic acid (TCA) can inhibit pathophysiologic features of AMD, as it prevented oxidative stress-induced disruption of tight junctions in primary human RPE cells and suppressed VEGF-induced cell migration and tube formation in rhesus macaque CECs [15].

The purpose of the current study was to determine if the glycine-conjugated bile acids glycocholic acid (GCA), glycodeoxycholic acid (GDCA), and glycoursodeoxycholic acid (GUDCA) can protect RPE tight junction integrity against oxidative stress-mediated damage and if they can inhibit VEGF-induced of angiogenesis in CECs.

## 2. Materials and Methods

### 2.1. Cell Culture

Human primary RPE cells (HRPEpiC) were purchased from ScienCell (Carlsbad, CA, USA) and maintained in epithelial cell medium (ScienCell). Rhesus macaque choroidal endothelial cells (RF/6A) were purchased from American Type Culture Collection (Manassas, VA, USA). RF/6A cells were maintained in Dulbecco’s Modified Eagle Medium (DMEM, Gibco, Invitrogen, Carlsbad, CA, USA) supplemented with 10% fetal bovine serum (FBS, Gibco, Invitrogen), 100 units/mL penicillin, and 100 µg/mL streptomycin (Gibco, Invitrogen,). RPE cells were cultured for two weeks prior to experiments, and experiments were conducted in epithelial cell medium. CECs were serum-starved in DMEM with 2% FBS overnight prior to experiments, and all RF/6A experiments were performed in DMEM with 0.5% FBS. Cells were incubated at 37 °C, 5% CO_2_, 20.9% O_2_, and 95% relative humidity. Sodium glycocholate (Sigma-Aldrich #G7132, St. Louis, MO, USA), sodium glycodeoxycholate (Calbiochem, Millipore #361311, Burlington, MA, USA), and sodium glycoursodeoxycholate (Toronto Research Chemicals #G679545, Toronto, ON, Canada) were dissolved in water to make 10 mM stock solutions for use in experimental assays.

### 2.2. HRPEpiC Trans-Epithelial Electrical Resistance Assay

HRPEpiC cells were seeded at a density of 2.0 × 10^5^ on fibronectin-coated Transwell inserts (12 mm, 0.4 µm pore size, Corning) and grown in epithelial medium supplemented with 2% FBS [16] for 21 days to allow cells to achieve a confluent monolayer and form junctional complexes as confirmed by stabilized baseline TEER measurements. Cells were treated for 48 h with 300 µM paraquat to induce oxidative stress or paraquat with 10, 50, 100, 200, or 500 µM GCA, GDCA, or GUDCA to assay the bile acids’ ability to protect tight junction function against oxidative stress, as previously described [15]. TEER was measured at 0 and 48 h in three standard fields for each sample. TEER values for each sample were averaged, and untreated cells served as a control.

### 2.3. Immunostaining of HRPEpiC Tight Junctions

HRPEpiC cells were allowed to achieve a confluent monolayer and form junctional complexes. Cells were treated with 300 µM paraquat alone or with 10, 50, 100, 200, or 500 µM GCA, GDCA, or GUDCA to observe tight junction structure via localization of tight junction protein ZO-1, as previously described [15]. Briefly, cells were treated with paraquat and bile acid for 48 h and fixed with −20 °C methanol. Cells were then blocked in 2% BSA in PBS with 0.05% Tween20 (PBST) for one hour at room temperature, followed by a 1-h incubation with primary antibody directed against ZO-1 (Invitrogen, ThermoFisher, #61-7300) (1:200) in PBST at room temperature. Secondary antibody AlexaFluor 488 (ThermoFisher) was added and incubated for 1 h, protected from light. Slides were mounted in Fluoromont-G (SouthernBiotech, Birmingham, AL, USA), which contains DAPI for nuclear counterstaining.

### 2.4. RF/6A Cell Proliferation Assay

RF/6A cells were plated in triplicate at a density of 1.5 × 10^4^ in a 96 well plate and treated with 100 ng/mL VEGF alone or in conjunction with 10, 50, 100, 200, or 500 µM GCA, GDCA, or GUDCA for 24 h. Untreated cells served as the control. Cell proliferation was measured using a BrdU assay according to the manufacturer’s instructions (Cell Signaling Technology, Catalog # 6813S, Danvers, MA, USA). Five hours after treating cells with VEGF and bile acid, BrdU was added to each well. Following a 24-h incubation, cells were fixed and probed with the BrdU detection antibody and HRP-conjugated secondary antibody. Absorbance was measured at 450 nm. Absorbance values for each sample were averaged and normalized to the control to adjust for variation in absorbance values between experiments.

### 2.5. RF/6A Transwell Cell Migration Assay

RF/6A cells in 100 µL serum-free medium were seeded at a density of 1.0 × 10^5^ cells onto Transwell inserts (6.5 mm, pore size 8.0 µm; Costar, Corning, NY, USA) coated with 20 µg/mL fibronectin (upper chamber). Wells of a 24 well plate served as the lower chamber and contained 600 µL serum-free medium supplemented with 100 ng/mL VEGF to act as a chemoattractant and 0, 10, 50, 100, 200, or 500 µM GCA, GDCA, or GUDCA. Cells were allowed to migrate through the pores of the Transwell insert from upper to lower chamber for 24 h. The negative control contained only serum-free medium in the lower chamber. Following the 24-h incubation, cells that had not migrated were removed from the insert with a cotton swab. Cells that had migrated through the membrane (and were adherent to the bottom of the membrane) were fixed with 4% paraformaldehyde at room temperature for 5 min and stained with 0.1% crystal violet at room temperature for 5 min. The inserts were washed with ddH_2_O and dried with a cotton swab and by allowing to stand at room temperature for 10 min [17]. Three standard fields of each insert were photographed, and cells from each field were counted.

### 2.6. RF/6A Cell Tube Formation Assay

RF/6A cells were seeded at a density of 1.0 × 10^5^ onto polymerized growth reduced Matrigel and treated with 100 ng/mL VEGF alone or in conjunction with 10, 50, 100, 200, or 500 µM GCA, GDCA, or GUDCA. Cells were allowed to form tubes for five hours, after which, five standard fields from each well were photographed. Tube lengths were measured using ImageJ (NIH), and measurements for each sample were averaged.

### 2.7. Statistical Analysis

TEER measurements for paraquat-treated RPE cells were compared to untreated RPE cells using a two-tailed t-test to verify that paraquat induced functional disruption of tight junctions. To test if bile acids can rescue RPE cells from paraquat-induced TEER reduction, TEER measurements from RPE cells treated with paraquat and GCA, GDCA, or GUDCA were compared to RPE cells treated with paraquat alone using a one-way ANOVA with a Tukey’s Honest Significance Difference post hoc test. VEGF-treated CECs were compared to untreated CECs using a two-tailed *t*-test to verify that VEGF induced proliferation, migration, and tube formation in these cells. To test if bile acids could inhibit VEGF-induced CEC proliferation, migration, and tube formation, values from each assay were compared using a one-way ANOVA with a Tukey’s Honest Significant Difference post hoc test. CECs treated with VEGF in conjunction with GCA, GDCA, or GUDCA were compared to CECs treated with VEGF alone for statistical significance. A *p*-value < 0.05 was considered significant for all assays.

## 3. Results

### 3.1. GCA, GDCA, and GUDCA Protect RPE Tight Junctions against Oxidative Stress

**Figure 1:**
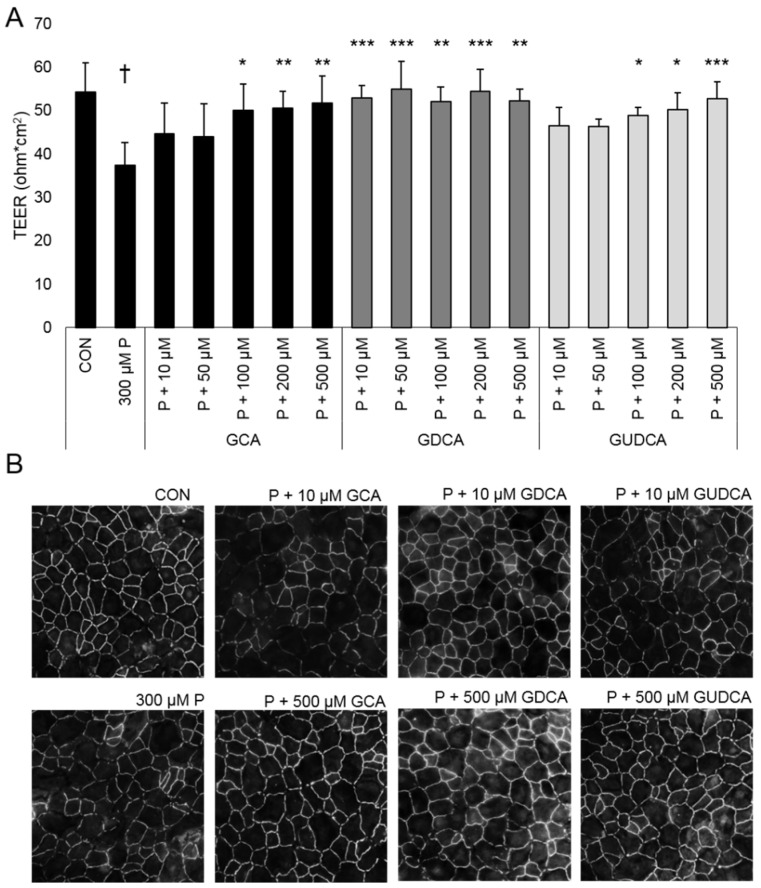
Glycine-conjugated bile acids protect RPE tight junctions against paraquat-induced oxidative stress. Primary RPE cells (HRPEpiC) were grown to confluence and treated with GCA, GDCA, or GUDCA to evaluate their ability to protect tight junction function and structure from paraquat-induced oxidative stress. (**A**) HRPEpiC cells were treated with 300 µM paraquat (P) or paraquat with GCA, GDCA, or GUDCA at 10, 50, 100, 200, or 500 µM for 48 h. Untreated cells served as a control. TEER measurements (ohm*cm^2^) at 48 h are presented as mean ± standard deviation (n = 5). †: *p* = 0.0029 compared to untreated control; *: *p* < 0.05 compared to paraquat alone; **: *p* < 0.01 compared to paraquat alone; ***: *p* < 0.001 compared to paraquat alone. (B) Immunofluorescent staining of ZO-1 in cells treated with 300 µM paraquat or paraquat with GCA, GDCA, or GUDCA at 10 or 500 µM.

To determine whether GCA, GDCA, and GUDCA could protect RPE cells against tight junction disruption caused by paraquat-induced oxidative stress, HRPEpiC cells were grown to confluence and treated with 300 µM paraquat to destabilize tight junctions. This treatment resulted in a 31.0% reduction in TEER (*p* = 0.0029), indicating a significant paraquat-induced decrease in tight junction function (Figure 1A). Each of the three bile acids was then tested at concentrations of 10, 50, 100, 200, and 500 µM for the ability to protect against the paraquat-induced decrease in TEER. GCA protected cells against TEER reduction at concentrations of 100 (*p* = 0.013), 200 (*p* = 0.0088), and 500 µM (*p* = 0.0023). GDCA abrogated paraquat-induced TEER reduction at all concentrations tested (10 µM, *p* = 6.9 × 10^−4^; 50 µM, *p* = 6.4 × 10^−5^; 100 µM, *p* = 0.0017; 200 µM, *p* = 1.0 × 10^−4^; and 500 µM, *p* = 0.0015). Finally, GUDCA preserved TEER in paraquat treated cells at 100 (*p* = 0.042), 200 (*p* = 0.012), and 500 µM (*p* = 7.5 × 10^−4^) concentrations.

To visualize tight junction disruption in RPE cells caused by oxidative stress, HRPEpiC cells were treated with 300 µM paraquat and immunostained with an antibody directed against the tight junction protein ZO-1. Cells treated with paraquat demonstrated decreased ZO-1 membrane localization compared to untreated cells, signifying structural disruption of tight junctions (Figure 1B). HRPEpiC cells were treated with paraquat in combination with 10–500 µM GCA, GDCA, or GUDCA to determine whether any of the bile acids could prevent tight junction disruption. Cells treated with paraquat and GCA showed disruption of ZO-1 staining at 10 and 50 µM but preservation of ZO-1 membrane localization at 100, 200, and 500 µM. GDCA preserved ZO-1 membrane localization at all five concentrations tested. Finally, GUDCA demonstrated preservation of ZO-1 membrane localization at 100, 200, and 500 µM in paraquat treated cells. Representative images of cells treated with 300 µM paraquat and 10 µM or 500 µM GCA, GDCA, and GUDCA are depicted in Figure 1B. Taken together, the TEER measurements and ZO-1 immunostaining demonstrate that GCA, GDCA, and GUDCA protect RPE tight junction structure and function against oxidative stress-induced damage.

### 3.2. GCA, GDCA, and GUDCA Do Not Inhibit VEGF-Induced CEC Proliferation

**Figure 2:**
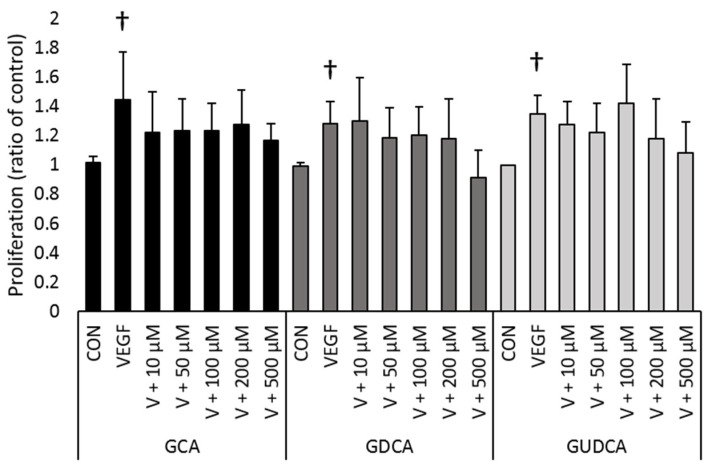
Glycine-conjugated bile acids do not significantly inhibit VEGF-induced CEC proliferation. RF/6A cell proliferation was induced with 100 ng/mL VEGF (V). Cells were treated with GCA, GDCA, or GUDCA (10, 50, 100, 200, or 500 µM) and their ability to inhibit cell proliferation was evaluated with a BrdU-based colorimetric assay. Absorbance levels were normalized to average absorbance levels of untreated control. Normalized absorbance levels of cells treated with VEGF were compared to untreated controls for each set of experiments (†: GCA, *p* = 0.023; GDCA, *p* = 0.0050; GUDCA, *p* = 0.0011). Normalized absorbance levels of cells treated with GCA, GDCA, or GUDCA were compared to normalized levels of cells treated with VEGF. No concentration of GCA, GDCA, or GUDCA significantly inhibited proliferation compared to cells treated with VEGF alone. Data are presented as mean ± standard deviation (n = 6).

The ability of GCA, GDCA, and GUDCA to inhibit VEGF-induced RF/6A proliferation was evaluated using a BrdU-based proliferation assay. RF/6A cells were treated with 100 ng/mL VEGF to stimulate cell proliferation. Cells treated with VEGF averaged 35.5% greater cell proliferation compared to untreated control cells (*p* < 0.023). To determine if any of the three bile acids could inhibit VEGF-induced cell proliferation, RF/6A cells were treated with 100 ng/mL VEGF and GCA, GDCA, or GUDCA at 10, 50, 100, 200, or 500 µM. None of the GCA concentrations inhibited proliferation compared to cells treated with VEGF alone (10 µM, *p* = 0.57; 50 µM, *p* = 0.63; 100 µM, *p* = 0.64; 200 µM, *p* = 0.81; 500 µM, *p* = 0.34). GDCA also did not inhibit cell proliferation at any concentration tested (10 µM, *p* = 1.0; 50 µM, *p* = 0.97; 100 µM, *p* = 0.99; 200 µM, *p* = 0.97; 500 µM, *p* = 0.073). Similarly, GUDCA showed no effect on VEGF-induced cell proliferation at any concentration (10 µM, *p* = 0.99; 50 µM, *p* = 0.90, 100 µM, *p* = 0.99; 200 µM, *p* = 0.75; 500 µM, *p* = 0.29). These data show that none of the three bile acids was able to inhibit VEGF-induced CEC proliferation at the concentrations assayed (Figure 2).

### 3.3. GCA and GUDCA Inhibit VEGF-Induced CEC Migration

**Figure 3:**
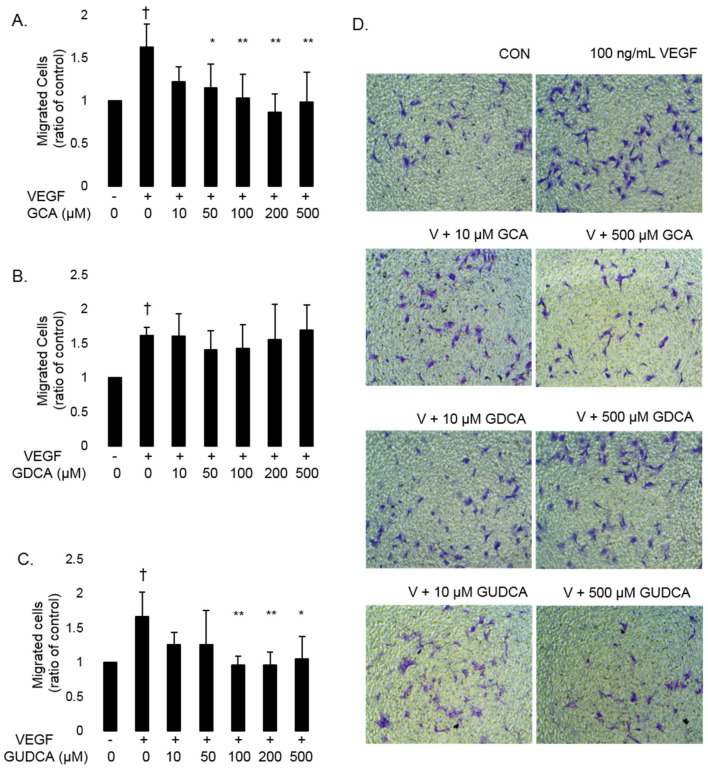
GCA and GUDCA inhibit VEGF-induced CEC migration. RF/6A cell migration was induced by treating cells with 100 ng/mL VEGF (V). Cells were treated with GCA, GDCA, or GUDCA (10, 50, 100, 200, or 500 µM) to evaluate their ability to inhibit cell migration. The number of migrated cells were counted from three standard fields and normalized to the average number of migrated cells in the untreated control. (**A**) VEGF induced cell migration compared to untreated controls (†: *p* = 0.0025). GCA did not inhibit migration at 10 µM and significantly inhibited VEGF-induced cell migration at 50, 100, 200, and 500 µM. (**B**) VEGF induced migration compared to untreated controls (†: *p* = 5.64 × 10^−5^). GDCA did not inhibit migration at any concentration tested. (**C**) VEGF induced migration compared to untreated controls (†: *p* = 0.0056). GUDCA did not inhibit migration at 10 or 50 µM and inhibited VEGF-induced migration at 100, 200, and 500 µM. Data are presented as mean ± standard deviation (*n* = 6). *: *p* < 0.05; **: *p* < 0.01 for bile acid-treated cells compared to VEGF-treated cells. (**D**) Representative images of cell migration of untreated controls (CON), cells treated with 100 ng/mL VEGF (V), and cells treated with VEGF plus 10 µM and 500 µM concentrations of GCA, GDCA, and GUDCA.

RF/6A endothelial cell migration was induced with VEGF, and cells were treated with VEGF in conjunction with 10, 50, 100, 200, and 500 µM GCA, GDCA, and GUDCA to determine if any of these bile acids could inhibit VEGF-induced cell migration. In the Transwell migration assay, VEGF-treated cells had an average of 63.7% more migrating cells than untreated control cells (*p* < 0.0056). GCA inhibited VEGF-induced cell migration at 50 (*p* = 0.047), 100 (*p* = 0.0067), 200 (*p* = 3.4 × 10^−4^), and 500 µM (*p* = 0.0030). None of the GDCA concentrations tested inhibited VEGF-induced cell migration compared to cells treated with VEGF alone (10 µM, *p* = 1.0; 50 µM, *p* = 0.89; 100 µM, *p* = 0.94; 200 µM, *p* = 1.0; 500 µM, *p* = 1.0). VEGF-induced CEC migration was inhibited by GUDCA at 100 (*p* = 0.0044), 200 (*p* = 0.0049), and 500 µM (*p* = 0.018). These results show that GCA and GUDCA can inhibit VEGF-induced CEC migration, while GDCA has no significant effect on VEGF-induced CEC migration (Figure 3).

### 3.4. GCA and GUDCA Inhibit VEGF-Induced CEC Tube Formation

**Figure 4:**
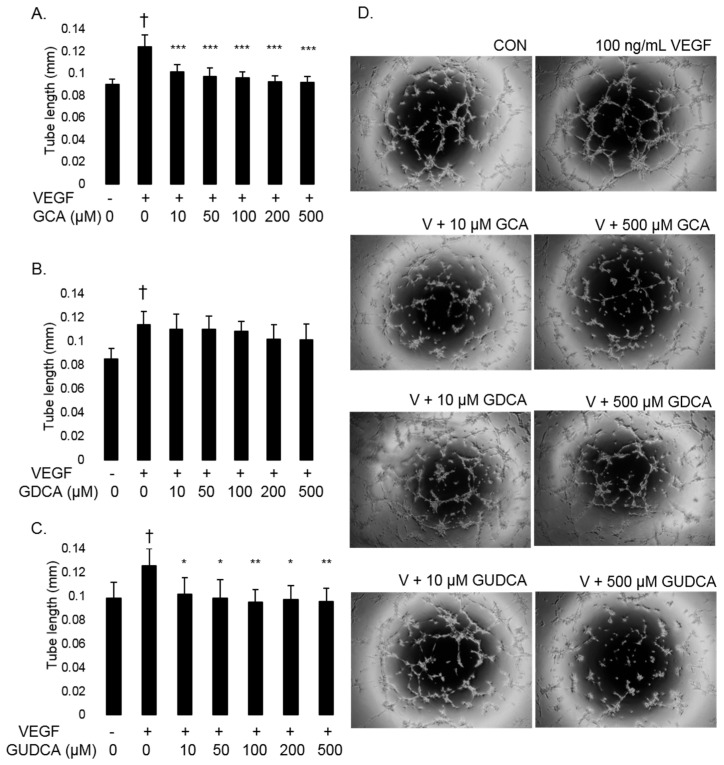
GCA and GUDCA inhibit VEGF-induced tube formation in RF/6A cells. RF/6A tube formation was induced by treating cells with 100 ng/mL VEGF (V). Cells were treated with GCA, GDCA, or GUDCA (10, 50, 100, 200, or 500 µM) to evaluate their ability to inhibit tubulogenesis. (**A**) VEGF induced tube formation compared to untreated cells (†: *p* = 1.7 × 10^−4^). RF/6A cells treated with VEGF and GCA resulted in significantly shorter tube length at 10, 50, 100, 200, and 500 µM. (**B**) VEGF induced tube formation compared to untreated cells (†: *p* = 6.3 × 10^−4^). No concentration of GDCA resulted in significantly shorter tubes compared to cells treated with VEGF alone. (**C**) VEGF induced tube formation compared to untreated cells (†: *p* = 0.0074). All concentrations of GUDCA resulted in significantly shorter tubes compared to cells treated with VEGF alone. Data are presented as average tube length ± standard deviation (*n* = 6). *: *p* < 0.05; **: *p* < 0.01; ***: *p* < 0.001 for bile acid-treated cells compared to VEGF-treated cells. (**D**) Representative images of tube formation of untreated controls (CON), cells treated with 100 ng/mL VEGF (V), and cells treated with VEGF plus 10 µM and 500 µM concentrations of GCA, GDCA, and GUDCA.

The ability of GCA, GDCA, and GUDCA to inhibit VEGF-induced RF/6A tube formation was evaluated using a Matrigel-based tube formation assay. Treating RF/6A cells with 100 ng/mL VEGF resulted in 33.0% longer tubes compared to untreated controls (*p* < 0.0074). RF/6A cells were treated with VEGF in conjunction with GCA, GDCA, or GUDCA at 10, 50, 100, 200, and 500 µM to determine if any of these bile acids could block VEGF-induced tube formation. GCA inhibited VEGF-induced tube formation at 10 (*p* = 4.6 × 10^−5^), 50 (*p* = 2.9 × 10^−6^), 100 (*p* = 1.1 × 10^−6^), 200 (*p* = 1.0 × 10^−7^), and 500 µM (*p* = 1.0 × 10^−7^) compared to cells treated with VEGF alone. No concentrations of GDCA tested significantly reduced tube length compared to cells treated with VEGF alone (10 µM, *p* = 0.99; 50 µM, *p* = 0.99; 100 µM, *p* = 0.96; 200 µM, *p* = 0.45; 500 µM, *p* = 0.42). VEGF-induced tube formation was inhibited by GUDCA at 10 (*p* = 0.039), 50 (*p* = 0.013), 100 (*p* = 0.0037), 200 (*p* = 0.0082), and 500 µM (*p* = 0.0051) compared to cells treated with VEGF alone. These data show that GCA and GUDCA inhibit VEGF-induced tube formation in CECs, while GDCA has no effect on tube formation (Figure 4).

## 4. Discussion

The current study demonstrates that the glycine-conjugated bile acids GCA, GDCA, and GUDCA structurally and functionally protect RPE tight junctions against oxidative stress in vitro, preserving both ZO-1 membrane localization and TEER. GCA and GUDCA, but not GDCA, inhibit VEGF-induced CEC angiogenesis as evidenced by in vitro cell migration and tube formation assays. None of the three bile acids significantly affected VEGF-induced CEC proliferation.

These results add to our previous finding that TCA protects RPE tight junction integrity and inhibits VEGF-induced CEC angiogenesis [15] in two ways. First, we have now shown that multiple bile acids are capable of inhibiting oxidative stress-induced RPE cell monolayer disruption and VEGF-induced CEC migration and tube formation in vitro, cellular features that have all been associated with advanced stages of AMD. This broadens our understanding of bile acid involvement in cellular signaling in the eye and expands the list of bile acids with potential therapeutic effects against AMD. Secondly, this study indicates that taurine conjugation is not required for bile acids to protect RPE cells from oxidative stress or to inhibit cellular mechanisms of VEGF-induced angiogenesis in CECs in vitro.

The fact that GCA, GDCA, and GUDCA differed in their ability to protect RPE tight junctions from oxidative stress and inhibit VEGF-induced CEC angiogenesis provides clues as to the mechanisms by which bile acids signal in these cells. Each of the three bile acids protected RPE tight junctions from paraquat-induced oxidative damage. GDCA preserved TEER at concentrations of 10–500 µM, while GCA and GUDCA maintained TEER at concentrations of 100 µM or higher. In CEC angiogenesis assays, the differences among the bile acids’ effects were even greater. GCA and GUDCA inhibited VEGF-induced cell migration and tubulogenesis, but GDCA had no significant effect on VEGF-treated CECs in any angiogenesis assay. The fact that different sets of glycine-conjugated bile acids protect RPE function and inhibit CEC angiogenesis leads us to hypothesize that these bile acids are activating different signaling pathways in the two cell types. Bile acids are known to activate a number of unique nuclear and membrane receptors [18], but which bile acid receptors are present in RPE cells and CECs is currently not well understood. Studies confirming bile acid receptor expression in RPE cells and CECs may provide further insight into the molecular pathways involved.

Both the structure of a bile acid’s steroid backbone and its conjugation can affect hydrophilicity, bile acid receptor binding, and cellular response [19]. These structural differences may help explain the variations we observed in cellular effects among the glycine-conjugated bile acids. The primary bile acids CA and CDCA are distinguished by their hydroxyl groups at carbons C3, C7, and C12, with CA containing a hydroxyl group in the α orientation (α-OH) at all three positions, while CDCA has an α-OH only at C3 and C7. Dehydroxylation of the 7α-OH in CA and CDCA forms the secondary bile acids deoxycholic acid (DCA) and lithocholic acid (LCA) [20]. UDCA is formed by epimerization of the 7α-OH of CDCA to 7β-OH [20]. CA, DCA, and UDCA are conjugated with glycine to form GCA, GDCA, and GUDCA, respectively. Thus, the primary molecular feature that distinguishes GDCA from GCA and GUDCA is that GDCA does not have the α-OH at C7. Since GDCA was the only bile acid we assayed that did not inhibit VEGF-induced CEC migration and tube formation, it may be that the 7α-OH is necessary for bile acids to inhibit CEC angiogenesis.

Paraquat induces oxidative stress [21], and paraquat and oxidative stress have been shown to disrupt epithelial cell tight junctions and ZO-1 expression in multiple types of epithelial cells, including RPE [22,23,24,25]. We showed here that GDCA preserved RPE tight junction function and structure at all concentrations tested, including as low as 10 µM, while GCA and GUDCA were protective only at concentrations of 100 µM or greater. This suggests that the 7α-OH may be important to bile acid signaling in RPE cells as well. LCA and CDCA have previously been reported to have opposite effects on tight junction integrity in the gastrointestinal epithelial barrier [26]. While CDCA induced oxidative stress, decreased TEER, and disrupted localization of the tight junction protein occludin, LCA restored TEER and attenuated oxidative stress caused by CDCA, but did not affect occludin localization and expression [26]. TUDCA has been found to decrease reactive oxygen species (ROS) production in ARPE-19 cells [10], and GUDCA has been shown to inhibit ROS in Barrett’s esophagus epithelial cells [27]. To our knowledge, the current study and our previous work with TCA [15] are the only studies investigating the relationship between RPE tight junctions and bile acids.

Kundu et al. evaluated the ability of non-conjugated bile acids to alter angiogenesis in in vitro and in vivo studies [28]. They found that the hydrophobic bile acids deoxycholic acid (DCA) and LCA inhibited tube formation and cell migration in human umbilical endothelial cells (HUVECs) in vitro, but LCA was cytotoxic to cells and could only be studied at lower, non-lethal concentrations. The hydrophilic bile acids CA and CDCA had differing effects on angiogenesis. CA had no effect on angiogenesis in vitro, while CDCA actually induced angiogenesis in vitro and in zebrafish [28]. Our studies have shown that taurine and glycine conjugates of CA inhibit in vitro CEC migration and tubulogenesis. While Kundu et al. reported that DCA inhibited migration and tube formation in HUVECs, we found that its glycine conjugate GDCA had no effect on CEC angiogenesis. Interestingly, the current study shows that CEC tubulogenesis is inhibited by even lower concentrations of GCA and GUDCA than we observed with TCA in our previous study.

In addition to increasing hydrophilicity, conjugation of bile acids with taurine or glycine increases solubility [29] and limits passive diffusion across the cell membrane [30]. The bile acid transporter OATP1B3 has been found to be expressed in the retina [31] and has been shown to prefer taurine- and glycine-conjugated bile acids over unconjugated bile acids [30]. Additionally, conjugated bile acids have been shown to activate the bile acid receptors TGR5 and S1P2 [29]. At this point, it is not clear if taurine or glycine conjugation is superior in affecting bile acids’ ability to protect RPE cells and limit choroidal angiogenesis, and further investigation is warranted.

The current study is limited by its use of in vitro assays to study cellular processes that are typically associated with AMD pathology to study the ability of GCA, GDCA, and GUDCA to prevent features of AMD development. An immortalized macaque choroidal endothelial line was used for the angiogenesis assays, and it may not accurately model AMD conditions in vivo. However, the study offers new insight into the potential of glycine-conjugated bile acids as therapeutic agents in retinal diseases, particularly AMD. We report here, for the first time, the ability of GCA, GDCA, and GUDCA to protect RPE tight junction integrity and of GCA and GUDCA to inhibit CEC angiogenesis. While previous bile acid studies have focused on the effects of UDCA or TUDCA in retinal models of photoreceptor degeneration, we have shown in this study and our previous work the therapeutic potential of GCA, GDCA, GUDCA, and TCA. A recent study found orally administered UDCA could cross the BRB in patients with retinal detachment, and subretinal fluid collected during vitrectomy from patients with UDCA oral supplementation protected rat retinas from retinal degeneration in an ex vivo model [32]. Such studies, along with the results presented here suggest that bile acids deserve further investigation as therapeutic agents for AMD.

In this study, GCA, GDCA, and GUDCA have demonstrated promising results as protective agents against progression to advanced stages of AMD. More studies are needed to understand the molecular mechanisms and signaling pathways through which GCA, GDCA, and GUDCA protect RPE cells and inhibit angiogenesis in choroidal endothelial cells.
